# Predictive Value of Machine Learning for Recurrence of Atrial Fibrillation after Catheter Ablation: A Systematic Review and Meta-Analysis

**DOI:** 10.31083/j.rcm2411315

**Published:** 2023-11-16

**Authors:** Xingman Fan, Yanyan Li, Qiongyi He, Meng Wang, Xiaohua Lan, Kaijie Zhang, Chenyue Ma, Haitao Zhang

**Affiliations:** ^1^Graduate School, Hebei North University, 075000 Zhangjiakou, Hebei, China; ^2^Department of Cardiology, Air Force Medical Center, Air Force Medical University, PLA,100142 Beijing, China; ^3^Air Force Clinical medical college, Fifth Clinical College of Anhui Medical University, 230032 Hefei, Anhui, China

**Keywords:** atrial fibrillation, machine learning, recurrence, prediction model, ablation, meta-analysis

## Abstract

**Background:**

Accurate detection of atrial fibrillation (AF) recurrence after catheter ablation is crucial. In this study, we aimed to conduct a systematic review of machine-learning-based recurrence detection in the relevant literature.

**Methods:**

We conducted a comprehensive search of PubMed, Embase, Cochrane, and Web of Science databases from 1980 to December 31, 2022 to identify studies on prediction models for AF recurrence risk after catheter ablation. We used the prediction model risk of bias assessment tool (PROBAST) to assess the risk of bias, and R4.2.0 for meta-analysis, with subgroup analysis based on model type.

**Results:**

After screening, 40 papers were eligible for synthesis. The pooled concordance index (C-index) in the training set was 0.760 (95% confidence interval [CI] 0.739 to 0.781), the sensitivity was 0.74 (95% CI 0.69 to 0.77), and the specificity was 0.76 (95% CI 0.72 to 0.80). The combined C-index in the validation set was 0.787 (95% CI 0.752 to 0.821), the sensitivity was 0.78 (95% CI 0.73 to 0.83), and the specificity was 0.75 (95% CI 0.65 to 0.82). The subgroup analysis revealed no significant difference in the pooled C-index between models constructed based on radiomics features and those based on clinical characteristics. However, radiomics based showed a slightly higher sensitivity (training set: 0.82 *vs.* 0.71, validation set: 0.83 *vs.* 0.73). Logistic regression, one of the most common machine learning (ML) methods, exhibited an overall pooled C-index of 0.785 and 0.804 in the training and validation sets, respectively. The Convolutional Neural Networks (CNN) models outperformed these results with an overall pooled C-index of 0.862 and 0.861. Age, radiomics features, left atrial diameter, AF type, and AF duration were identified as the key modeling variables.

**Conclusions:**

ML has demonstrated excellent performance in predicting AF recurrence after catheter ablation. Logistic regression (LR) being the most widely used ML algorithm for predicting AF recurrence, also showed high accuracy. The development of risk prediction nomograms for wide application is warranted.

## 1. Introduction

As the global population ages at an accelerated rate, atrial fibrillation (AF) has emerged as one of the cardiovascular diseases with the highest incidence in the 21st Century [1]. In the United States alone, at least 3 to 6 million individuals are currently suffering from AF. Early rhythm control can significantly reduce the risk of cardiovascular adverse events among AF patients [2]. Two common rhythm control methods used in clinical practice include (1) catheter ablation treatment and (2) antiarrhythmic drug therapy [3, 4]. The catheter ablation treatment has been shown to outperform drug therapy, as it aids patients in recovering from sinus rhythm [3, 5] and improves their quality of life during early disease progression [6]. However, it’s important to note that AF reoccurs in approximately a third of patients undergoing catheter ablation [7]. Therefore, it is important to assess AF recurrence following ablation to develop primary prevention strategies. Although ${\mathrm{CHADS}}_{2}$, ${\mathrm{CHA}}_{2}$${\mathrm{DS}}_{2}$-VASc, and $R_{2}$${\mathrm{CHADS}}_{2}$ scores can be used to predict AF recurrence after catheter ablation, their predictive accuracy remains unsatisfactory [8]. Consequently, it remains to be proven if the prediction models can truly improve patient prognosis.

Recent advances in artificial intelligence, statistics, and machine learning (ML) have gradually found new applications in clinical settings, including disease diagnosis and prognosis [9, 10, 11]. In this context, some investigators have utilized ML to identify risk factors related to the early recurrence of AF following catheter ablation, and to construct prognostic models to maximize clinical outcomes [12, 13]. However, prediction accuracy remains controversial since ML covers many mathematical methods, variables, and models. Therefore, this study aimed to explore the predictive performance of ML for AF recurrence following catheter ablation, and comprehensively summarize modeling variables, thus promoting the development of risk stratification tools in the field.

## 2. Methods

### 2.1 Study Registration 

This systematic review was conducted following the requirements of the preferred reporting items for systematic reviews and meta-analyses (PRISMA2020) (**Supplementary Table 1**) [14], and registered via PROSPERO (ID: CRD42023401497).

### 2.2 Inclusion and Exclusion Criteria

#### 2.2.1 Inclusion Criteria

(1) Studies occurred in patients diagnosed with AF who underwent catheter ablation.

(2) The observed outcome event was AF recurrence, and a ML prediction model was constructed.

(3) Different studies may apply the same data set to different ML models, and these models may have different variables. Therefore, different studies on ML algorithms published based on the same data set were included in this systematic review.

(4) Studies without an independent validation set were included in this systematic review.

(5) Original study type includes cohort studies, randomized controlled trials (RCTs), case-control studies, cross-sectional studies, case-cohort studies, and nested case-control studies.

(6) Literature reported in English.

#### 2.2.2 Exclusion Criteria

(1) Studies with significant flaws in diagnosing AF or recurrence of AF.

(2) Only the risk factors were analyzed, and no complete ML model was constructed.

(3) Studies lacking the following outcome measures in assessing the accuracy of ML models: Roc, C-statistics, concordance index (C-index), sensitivity, specificity, accuracy, recovery rate, accuracy rate, confusion matrix, diagnostic fourfold table, F1 score, and calibration curve.

(4) Studies only on the validation of a maturity scale.

(5) Studies on the accuracy of single-factor prediction.

(6) Meta-analyses, reviews, guidance, expert opinions, or articles of similar nature.

### 2.3 Data Sources and Search Strategy 

PubMed, Embase, Web of Science, and Cochrane databases were searched from 1980 to December 31, 2022, by combining the subject terms and subheadings of “atrial fibrillation”, “recurrence” and “machine learning”. The complete search strategy is shown in **Supplementary Table 2**.

### 2.4 Study Selection and Data Extraction 

All retrieved literature was imported into Endnote. After removing duplications, titles and abstracts were reviewed to exclude irrelevant studies. Subsequently, the full texts of the studies selected in the initial screening were downloaded and read to select eligible original studies. A data extraction table was prepared in advance to record the following data: study types (e.g., cohort studies, cross-sectional studies), study characteristics (e.g., author, year, title, and author’s country), study groups (e.g., total sample size, number of relapsed cases, total number of cases in the training set, number of recurrent cases in the training set, number of recurrent cases in the validation set, and the total number of cases in the validation set), ablation type, follow-up time, definition of blank period, definition of AF recurrence, method of generating the validation set, overfitting method, missing value treatment method, variable screening method, model type, and modeling variables.

The literature screening and data extraction were independently conducted by two investigators (XF and XL), with a cross-check conducted following completion. In the event of any disagreements or uncertainties regarding the eligibility of a particular study, another reviewer (YL) was consulted for resolution.

### 2.5 Risk of Bias in the Included Studies

The prediction model risk of bias assessment tool (PROBAST) [15] was used to assess the risk of bias in the original studies included. This tool included a total of 20 questions organized across four domains (participators, predictors, outcomes, and statistical analysis). Each question can be answered as Yes/Probably Yes, No/Probably No, or No Information. If a domain included at least one question answered with No or Probably No, it was considered to have a high bias risk. A domain was considered low risk if the answers to all questions were Yes or Probably Yes. The overall bias risk was considered low if all domains were classified as low risk. Conversely, if at least one domain is considered high risk, the overall risk of bias is regarded as high.

To ensure accuracy, two investigators (XF and XL) independently conducted the risk of bias assessment based on PROBAST and cross-checked their results. In case of any disagreements, a third investigator (YL) would be asked for assistance in reaching a judgment.

### 2.6 Outcomes

The C-index was utilized as the outcome measure to reflect the overall accuracy of the model. However, in case of severe imbalance between relapsed and non-relapsed cases, the C-index may not reflect the true prediction accuracy of models for the recurrence risk. Therefore, our main outcome measures also included sensitivity and specificity, and the secondary outcome measure was the frequency of occurrence of pooled modeling variables.

### 2.7 Statistical Analysis

If C-index lacked a 95% confidence interval (CI) and standard error in the original study, the standard error was estimated through the by Debray *et al. * [16] calculation method. Given the differences in the variables included in each ML model and the inconsistency in the parameters, we utilized a random-effects model for the meta-analysis of the C-index.

In addition, a bivariate mixed-effects model was employed to assess the sensitivity and specificity of the meta-analysis. Functioning as a random effects model, it accounts for the correlation between sensitivity and specificity. During the meta-analysis process, sensitivity and specificity were analyzed based on the diagnostic fourfold table, which unfortunately were not reported in most of the original studies. To address this, we utilized the following two methods to calculate the diagnostic fourfold table: (1) Calculate the fourfold table using sensitivity, specificity, and precision in combination with the number of cases; (2) Extract the sensitivity and specificity according to the best Youden’s index, and then calculate the fourfold table using the number of cases. The meta-analysis of the study was conducted using R4.2.0 (R development Core Team, Vienna, Austria, http://www.R-project.org).

## 3. Results

### 3.1 Study Selection

In total, 770 articles were identified from multiple databases. Out of these, 220 articles were duplicates and removed. After reviewing the titles and abstracts of the remaining 550 articles, 48 were selected for full-text assessment and downloaded.

**Fig. 1.**
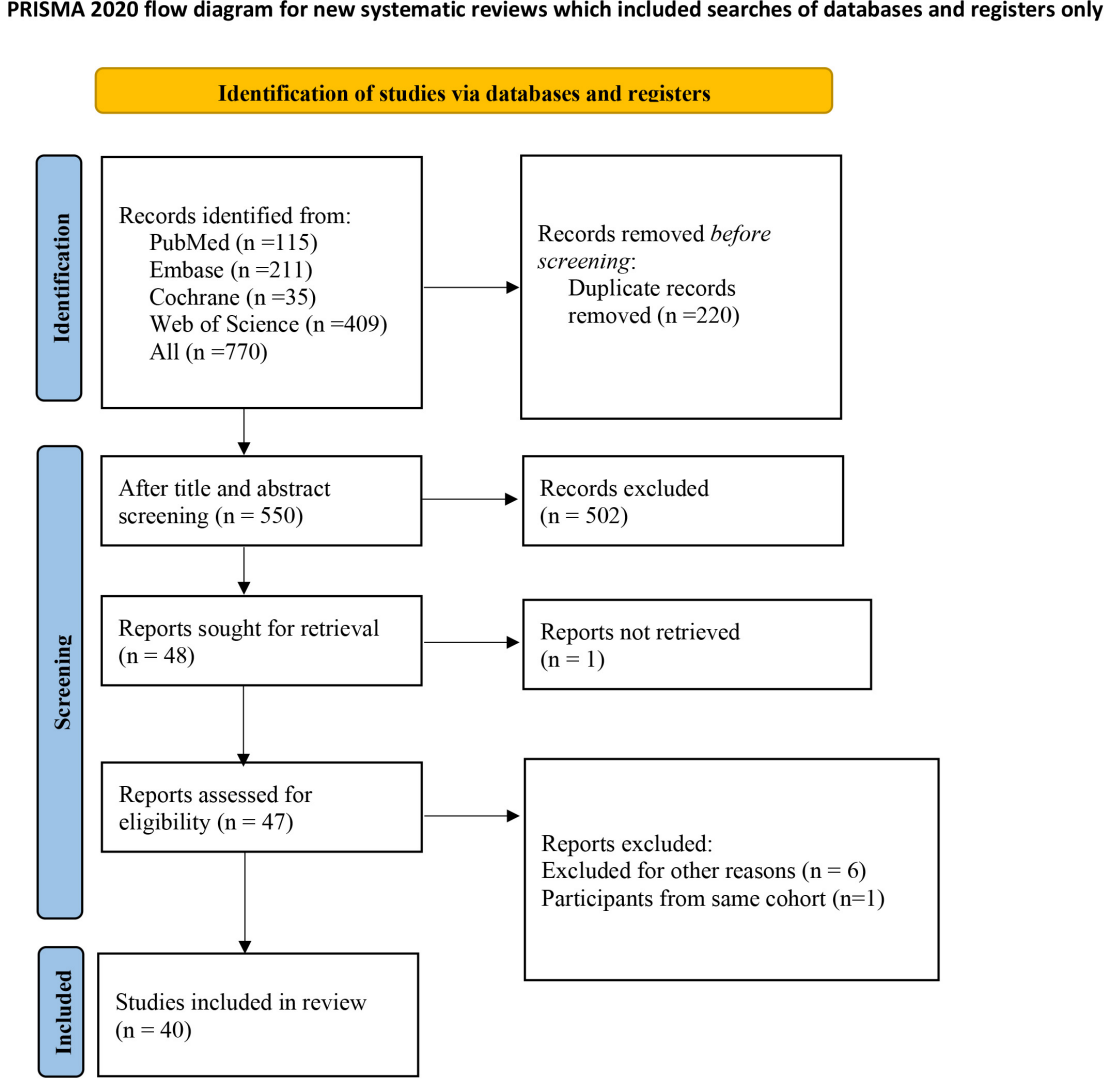
**PRISMA (preferred reporting items for systematic reviews and meta-analyses) flow diagram for study selection**.

Among them, one article was unavailable in full text, 6 articles were excluded for other reasons, and one article was deleted due to duplication of an identical cohort. Finally, 40 studies were included in this systematic review and meta-analysis [12, 17, 18, 19, 20, 21, 22, 23, 24, 25, 26, 27, 28, 29, 30, 31, 32, 33, 34, 35, 36, 37, 38, 39, 40, 41, 42, 43, 44, 45, 46, 47, 48, 49, 50, 51, 52, 53, 54, 55]. Fig. 1 displays the PRISMA flow chart outlining the study selection process.

### 3.2 Study Characteristics

This meta-analysis included 40 studies with a total of 16,251 AF patients receiving ablation treatment. From this total, 4930 (30.3%) patients experienced a recurrence of AF. The primary method used to record AF recurrence was body surface electrocardiogram (92.5%). Additionally, insertable loop recorders were used in 8 studies [12, 18, 26, 27, 29, 36, 49, 53], intracardiac electrogram was used in one study [52], and smart wearable devices were used in one study [27] (see attachment materials—** Supplementary Table 3** in detail). The 40 articles were published between 2015 to 2022, with 19 articles (45.2%) published in 2022 (see **Supplementary Fig. 1**). Among these, 31 were retrospective cohort studies. The majority of catheter ablation procedures were performed using radiofrequency ablation or cryoablation, and the average follow-up time ranged from 4 months to 120 months. Patients from the United States were represented in 6 studies [12, 17, 18, 19, 20, 21], Europe in 11 studies [22, 23, 24, 25, 26, 27, 28, 29, 30, 31, 32], and the Asia-Pacific region in 23 studies [33, 34, 35, 36, 37, 38, 39, 40, 41, 42, 43, 44, 45, 46, 47, 48, 49, 50, 51, 52, 53, 54, 55]. Regarding the ML algorithms, logistic regression was the most commonly used method for predicting AF recurrence after catheter ablation, accounting for 24 out of 40 studies (60%). The remaining studies utilized other ML methods, including K-nearest neighbor (KNN), logistic regression (LR), Cox proportional hazard model (COX), Cox proportional-hazards deep neural networks (DeepSurv), Adaptive boosting (Adaboost), support vector machine (SVM), convolutional neural networks (CNN), artificial neural network (ANN), extreme gradient boosting (XGBoost), random forest (RF), decision tree (DT), linear discriminant analysis (LDA). The characteristics of the included studies are detailed in **Supplementary Table 3**.

### 3.3 Modeling Variables

This study involved 93 predictors, with the top 5 are being age, radiomics features, left atrial diameter, type of AF, and AF duration. The remaining predictors include body mass index (BMI), sex, left ventricular ejection fraction (LVEF), hypertension, diabetes, and estimated glomerular filtration rate (eGFR) (see attachment materials—**Supplementary Table 3** for modeling variables in detail).

### 3.4 Risk of Bias in the Included Studies

**Fig. 2.**
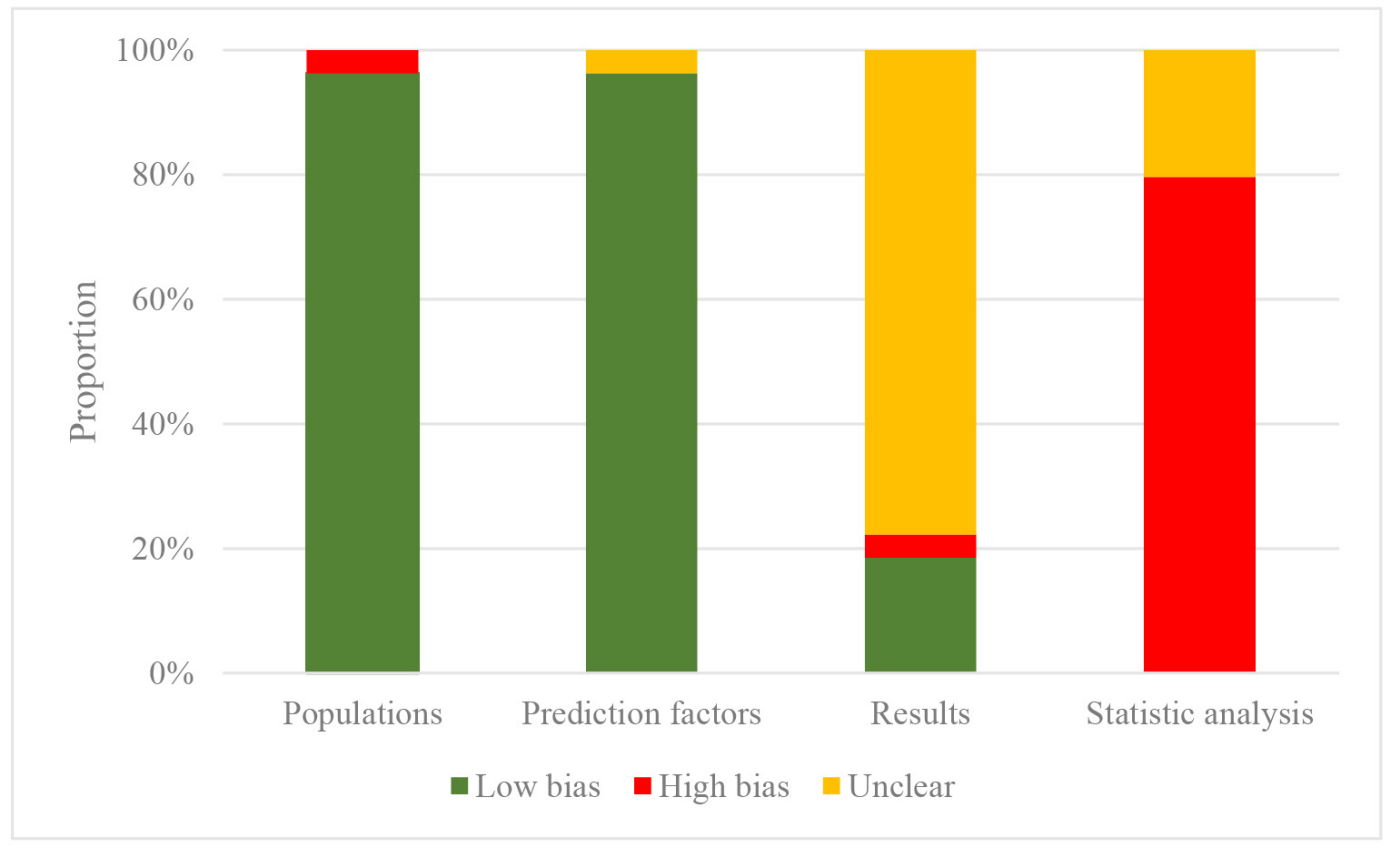
**Risk of Bias Assessment Result Included in the Machine Learning Model**.

The risk of bias and the overall applicability was assessed using the PROBAST checklist, which is provided in **Supplementary Table 1**. Details of the risk of bias and applicability for each model included in the study can be found in online **Supplementary Table 4**, and a summary of the bias risk is presented in Fig. 2.

Out of the 54 models identified of the 40 eligible studies, two models (3.7%) had high and moderate risks of bias in terms of participants and predictors, possibly because their study type, namely case-control design, makes it impossible to determine whether the source of participants is appropriate or whether the predictors were evaluated without knowing outcome data. The risk of bias in outcome was moderate in 42 models (77.8%). Regarding the statistical analysis, the underfitting process resulting from insufficient sample size or failure to overfit the prediction model led to a high risk of bias in 43 models.

### 3.5 Meta-Analysis

#### 3.5.1 Synthesized Results

The C-index of prediction modes for recurrent AF following catheter ablation treatment are shown in Table 1. Among the 40 included studies, the training set comprised a total of 48 models, with a pooled C-index of 0.760 (95% CI 0.739 to 0.781) calculated using the random effects model. The validation set consisted of 19 models, with a pooled C-index of 0.787 (95% CI 0.752 to 0.821). In the training set, the pooled fourfold tables of 40 models were either directly or indirectly reported, and the bivariable mixed model was utilized for the meta-analysis of sensitivity and specificity. The pooled sensitivity and specificity were 0.74 (95% CI 0.69 to 0.77) and 0.76 (95% CI 0.72 to 0.80), respectively. In the validation set, 15 models reported fourfold tables, and the bivariable mixed model was utilized for the meta-analysis of sensitivity and specificity. The pooled sensitivity and specificity were 0.78 (95% CI 0.73 to 0.83) and 0.75 (95% CI 0.65 to 0.82), respectively (Table 2).

**Table 1.** **Meta-analysis result of the C-index of machine learning in predicting the atrial fibrillation recurrence**.

Modeling variables	Model type	Training set		Validation set	
		n	C-index (95% CI)	n	C-index (95% CI)
Clinical characteristics					
	KNN	1	0.600 (0.549–0.651)		
	LR	19	0.775 (0.725–0.824)	5	0.777 (0.711–0.843)
	COX	9	0.735 (0.697–0.773)	3	0.820 (0.764–0.876)
	DeepSurv	1	0.730 (0.710–0.750)		
	Adaboost	1	0.711 (0.665–0.757)		
	SVM	1	0.638 (0.535–0.741)		
	CNN	2	0.864 (0.640–1.00)	1	0.861 (0.816–0.906)
	ANN	1	0.766 (0.678–0.854)		
	XGBoost	1	0.608 (0.503–0.713)		
	RF	1	0.718 (0.674–0.762)	1	0.721 (0.679–0.763)
	DT	1	0.599 (0.547–0.651)		
	Overall	38	0.751 (0.729–0.773)	10	0.794 (0.745–0.842)
Radiomics features					
	RF	2	0.717 (0.521–0.913)	1	0.870 (0.815–0.925)
	KNN	1	0.660 (0.554–0.766)	1	0.700 (0.589–0.811)
	LR	3	0.848 (0.729–0.967)	2	0.863 (0.777–0.948)
	XGBoost	2	0.766 (0.705–0.827)		
	SVM	1	0.850 (0.774–0.926)	3	0.713 (0.650–0.775)
	CNN	1	0.859 (0.796–0.922)		
	DT			1	0.630 (0.512–0.748)
	LDA			1	0.700 (0.572–0.827)
	Overall	10	0.793 (0.734–0.853)	9	0.779 (0.728–0.829)
All models					
	COX	9	0.735 (0.697–0.773)	3	0.820 (0.764–0.876)
	DeepSurv	1	0.730 (0.710–0.750)		
	CNN	3	0.862 (0.688–1.000)	1	0.861 (0.816–0.906)
	LR	22	0.785 (0.737–0.833)	7	0.804 (0.735–0.872)
	XGBoost	3	0.718 (0.621–0.816)		
	DT	1	0.599 (0.547–0.651)	1	0.630 (0.512–0.748)
	KNN	2	0.611 (0.565–0.657)	1	0.700 (0.589–0.811)
	AdaBoost	1	0.711 (0.665–0.757)		
	RF	3	0.725 (0.632–0.818)	2	0.794 (0.648–0.940)
	SVM	2	0.747 (0.539–0.955)	3	0.713 (0.650–0.775)
	ANN	1	0.766 (0.678–0.854)		
	LDA			1	0.700 (0.572–0.827)
	Overall	48	0.760 (0.739–0.781)	19	0.787 (0.752–0.821)

Abbreviations: 95% CI, 95% confidence interval; KNN, K-nearest neighbor; LR, logistic regression; COX, Cox proportional hazard model; DeepSurv, Cox proportional-hazards deep neural networks; Adaboost, adaptive boosting; SVM, support vector machine; CNN, convolutional neural network; ANN, artificial neural network; XGBoost, extreme gradient boosting; RF, random forest; DT, decision tree; LDA, linear discriminant analysis.

**Table 2.** **Meta-analysis result of the sensitivity and specificity of machine learning in predicting the atrial fibrillation recurrence**.

Modeling variables	Model type	Training set			Validation set		
		n	Sen (95% CI)	Spe (95% CI)	n	Sen (95% CI)	Spe (95% CI)
Clinical characteristics							
	KNN	1	0.58	0.57			
	LR	17	0.72 (0.66–0.77)	0.78 (0.71–0.83)	5	0.72 (0.63–0.80)	0.89 (0.73–0.96)
	COX	7	0.71 (0.60–0.80)	0.78 (0.71–0.83)	2	0.66–0.80	0.74–0.83
	Adaboost	1	0.64	0.70			
	SVM	1	0.62	0.66			
	CNN	1	0.92	0.94	1	0.80	0.79
	ANN	1	0.75	0.78			
	XGBoost	1	0.62	0.60			
	RF	1	0.73	0.64			
	DT	1	0.62	0.60			
	Overall	32	0.71 (0.67–0.76)	0.76 (0.72–0.80)	8	0.73 (0.66–0.79)	0.85 (0.75–0.92)
Radiomics features							
	KNN				1	0.79	0.54
	LR	4	0.89 (0.73–0.89)	0.76 (0.62–0.86)	2	0.80–0.82	0.60–0.85
	XGBoost	2	0.663–0.875	0.68–0.775			
	SVM	1	0.80	0.74	3	0.76–0.88	0.4–0.63
	CNN	1	0.87	0.87			
	DT				1	0.71	0.53
	Overall	8	0.82 (0.75–0.87)	0.76 (0.68–0.83)	7	0.83 (0.77–0.88)	0.64 (0.54–0.73)
All models							
	COX	7	0.71 (0.60–0.80)	0.78 (0.71–0.83)	2	0.66–0.80	0.74–0.83
	CNN	2	0.87–0.923	0.867–0.936	1	0.80	0.79
	LR	21	0.74 (0.68–0.79)	0.77 (0.72 –0.82)	7	0.73 (0.66–0.79)	0.85 (0.71–0.93)
	XGBoost	3	0.617–0.875	0.6–0.775			
	DT	1	0.62	0.60	1	0.71	0.53
	KNN	1	0.58	0.57	1	0.79	0.54
	AdaBoost	1	0.64	0.70			
	RF	1	0.73	0.64			
	SVM	2	0.617–0.8	0.662–0.74	3	0.76–0.88	0.4–0.63
	ANN	1	0.75	0.78			
	Overall	40	0.74 (0.69–0.77)	0.76 (0.72–0.80)	15	0.78 (0.73–0.83)	0.75 (0.65–0.82)

Abbreviations: 95% CI, 95% confidence interval; KNN, K-nearest neighbor; LR, logistic regression; COX, Cox proportional hazard model; Adaboost, adaptive boosting; SVM, support vector machine; CNN, convolutional neural network; ANN, artificial neural network; XGBoost, extreme gradient boosting; RF, random forest; DT, decision tree; Spe, specificity; Sen, sensitivity.

#### 3.5.2 Modeling Variables

The modeling variables were categorized into clinical characteristics or radiomics features for subgroup analysis. The results indicated there was no significant difference in the pooled C-index for either the training set or the validation set (training set: 0.751 *vs.* 0.793; validation set: 0.794 *vs.* 0.779). However, the prediction models constructed based on the radiomics features showed a higher sensitivity (training set: 0.82 [95% CI 0.75 to 0.87]; validation set: 0.83 [95% CI 0.77 to 0.88]) compared to those constructed from clinical characteristics (training set: 0.71 [95% CI 0.67 to 0.76]; validation set: 0.73 [95% CI 0.66 to 0.79]) in both the training and validation sets.

#### 3.5.3 Model Integrity

In the study, LR was the most commonly used ML algorithm, with 22 LR models and 7 LR models included in the training set and validation set, respectively. The pooled C-index for LR models was 0.785 (95% CI 0.737 to 0.833) in the training set and 0.804 (95% CI 0.735 to 0.872) in the validation set. Among non-LR models, the prediction models constructed based on the CNN algorithm showed the highest C-index, specificity, and sensitivity in both the training set and the validation set. Additionally, in the subgroup analysis by model type, two survival models (Cox and DeepSurv) were also reported. The training set C-index of Cox and DeepSurv were 0.735 (95% CI 0.697 to 0.773) and 0.730 (95% CI 0.710 to 0.750), respectively (Table 2).

## 4. Discussion

### 4.1 Summary of the Main Results/Findings

This meta-analysis aimed to assess the performance of ML models in predicting AF recurrence following ablation. The pooled C-index results of 54 models demonstrated the high accuracy of ML in predicting and recognizing AF recurrence. As a digital-driven method, ML allows continuous learning from data to refine the model using various statistical probability and optimization techniques. This feature presents significant opportunities for developing risk prediction models in cardiovascular research similar to the well-known Framingham Heart Study [56]. By developing risk models using ML, it becomes possible to classifying ablation-treated AF patients into different risk groups, which in turn, allows the formulation of personalized follow-up protocols based on the specific timing and populations. This approach can minimize overtreatment in low-risk populations and strike a better balance between the risk-benefit and cost-benefit in the screening of AF recurrence. Overall, ML holds promising potential in advancing the field of cardiovascular risk prediction and improving patient care.

We tested many methods of subgroup analysis to predict AF recurrence in patients after catheter ablation treatment. The traditional methods included logistic regression and Cox regression. Additionally, we explored the application of support vector machines, ensemble learning, artificial neural networks, deep learning, and other ML methods. Deep learning proved to be advantageous in image recognition and data processing, as it can convert low-level characteristic data into more abstract high-level characteristic data through layer-by-layer conversion. Based on the subgroup analysis results, the model constructed using the CNN algorithm by Yi-Ting Hwang *et al*. [52] demonstrated the highest C-index, specificity, and sensitivity. However, due to the limited number of models, it is essential to increase the sample size and conduct external validation to gather more robust risk assessment evidence. After considering the models constructed based on clinical characteristics and radiomics features, logistic regression emerged as the most commonly used method for predicting AF recurrence in patients after ablation treatment. It had the second-highest testing power compared to the CNN model in the training set and displayed the best specificity and sensitivity in the validation set. Given these advantages, logistic regression is expected to be effectively applied in developing nomograms based on clinical characteristics for predicting AF recurrence after ablation treatment.

The selection of variables in prediction models plays a critical role in their performance. Among the 54 models, 29 models included the age of AF patients receiving ablation treatment as a modeling variable. Age has been identified as the most likely risk factor for AF, more so than with sex, BMI, hypertension, and cardiac failure [57]. However, for AF patients receiving ablation treatment at different ages, there were no statistical differences in the AF recurrence rate [58, 59]. Another important modeling variable is radiomics features, formally proposed by Lambin in 2012 [60]. These high-dimensional features that not visible to the naked eye in medical digital images such as ultrasound, computed tomography (CT) and magnetic resonance imaging (MRI). However, they can be analyzed using high throughput programs. By transforming the image data of the region of interest (ROI) into high-resolution, exploitable spatial data using full-automatic or semi-automatic analysis methods, the accuracy of disease prediction, diagnosis, and prognosis estimation can be improved.

The subgroup analysis results showed no significant differences in the pooled C-index between the models constructed based on clinical characteristics and those based on radiomics features in either the training set or the validation set. This lack of difference may be due to data overfitting caused by excessive data extraction and decreased prediction performance resulting from inaccurate image segmentation [17, 30]. Nonetheless, prediction models constructed based on radiomics features exhibited higher sensitivity, which is clinically significant for predicting AF recurrence after ablation.

While several studies have highlighted the significance of genetic variation in AF within the context of genomics [61, 62], none of the studies included in this review used alleles related to AF recurrence after ablation as predictors for model development.

Moreover, most of the predictors in these models were came from the baseline data of AF patients before admission, such as BMI, eGFR and left atrial diameter. However, it’s important to note that these short-term risk factors are subject to change, and AF recurrence may be influenced by healthy habits after discharge. Unfortunately, these factors are rarely considered in the analysis of prediction models. A recent single-center, randomized controlled trial of symptomatic AF in obesity [63] demonstrated that weight control and enhanced management of risk factors in AF patients after discharge improved the long-term success rate of AF ablation.

### 4.2 Clinical Feasibility

As cross-disciplinary research in AI-medicine progresses, there is a growing focus on developing and validating prediction models based on ML algorithms for cardiovascular diseases [64, 65]. In this systematic review and meta-analysis, we combined the training set and the validation set (including both randomly acquired internal sampling results and a small number of external validation results) to assess the performance of ML in predicting AF recurrence in patients after ablation. The C-index results demonstrated high accuracy in both the training set (0.760 [0.739–0.781]) and the validation set (0.787 [0.752–0.821]) with similar prediction performance, and without overfitting. Among the top 5 risk predictors for AF recurrence after ablation, age, type of AF, and duration of AF are relatively easy to obtain, show small population differences, and high reproducibility, making them suitable for clinical use and popularization to a certain degree.

### 4.3 Strengths and Limitations

This systematic review represents the first attempt to assess the predictive accuracy of ML for AF recurrence after ablation, providing evidence for the promising prediction capacity of ML models in these patients. However, our study does have some limitations.

First, the ML models included in the review suffered from high bias due to the rigid assessment using PROBAST for bias risk. In terms of statistical methods, a model is considered low bias only if the events per variable (EPV) is larger than 20 and it has an independent validation set with more than 100 cases. However, this rule ignores certain rare diseases or particular research fields (radiomics). Therefore, we focused on prediction factors and results for studies with high bias.

Second, an essential aspect of ML is selecting effective modeling variables. To minimize the discrepancy in modeling variables, we conducted subgroup analysis based on clinical characteristics and radiomics features, which reduced the number of models in the analysis process.

Third, radiomics lacks a standardized operating procedure, resulting in multiple approaches for dividing new areas, extracting texture features, screening modeling features, and constructing models. Despite this variability, it is important to acknowledge and recognize its clinical application value.

Finally, it is worth noting that some models in the included studies lacked valid independent validation sets [25, 38, 39, 40]. Overcoming this limitation in systematic reviews of ML can be challenging. To address this issue, we combined the results of both the training set and the validation set to assess the value of ML by comparing their accuracy levels.

## 5. Conclusions

In conclusion, the ML method has shown high performance in predicting AF recurrence, making it a competitive and cost-effective approach to screening the AF recurrence after ablation. In the future, multi-center, large-sample clinical data sets can be established to develop the correlation nomogram for predicting AF recurrence after ablation based on LR. Additionally, to enhance the efficiency and feasibility of the model, future predictors should not only focus on the baseline data indicators of AF patients after ablation but also include radiomics features and post-discharge health habits of AF patients.

## Data Availability

All data generated or analyzed during this study are included in this published article or are available from the corresponding author on reasonable request.

## Supplementary Material

Supplementary material associated with this article can be found, in the online version, at https://doi.org/10.31083/j.rcm2411315.
