# Supplementary material for: Predictive Value of Machine Learning for Recurrence of Atrial Fibrillation after Catheter Ablation: A Systematic Review and Meta-Analysis
Source: Rev Cardiovasc Med. 2023 Nov 16;24(11):315. doi: 10.31083/j.rcm2411315 (PMC11272879; doi:10.31083/j.rcm2411315)
Supplement: Supplementary file 1 [file 2153-8174-24-11-315-s1.zip › 2153-8174-24-11-315-s1/Supplementary Material-V2.docx]

Supplementary materials


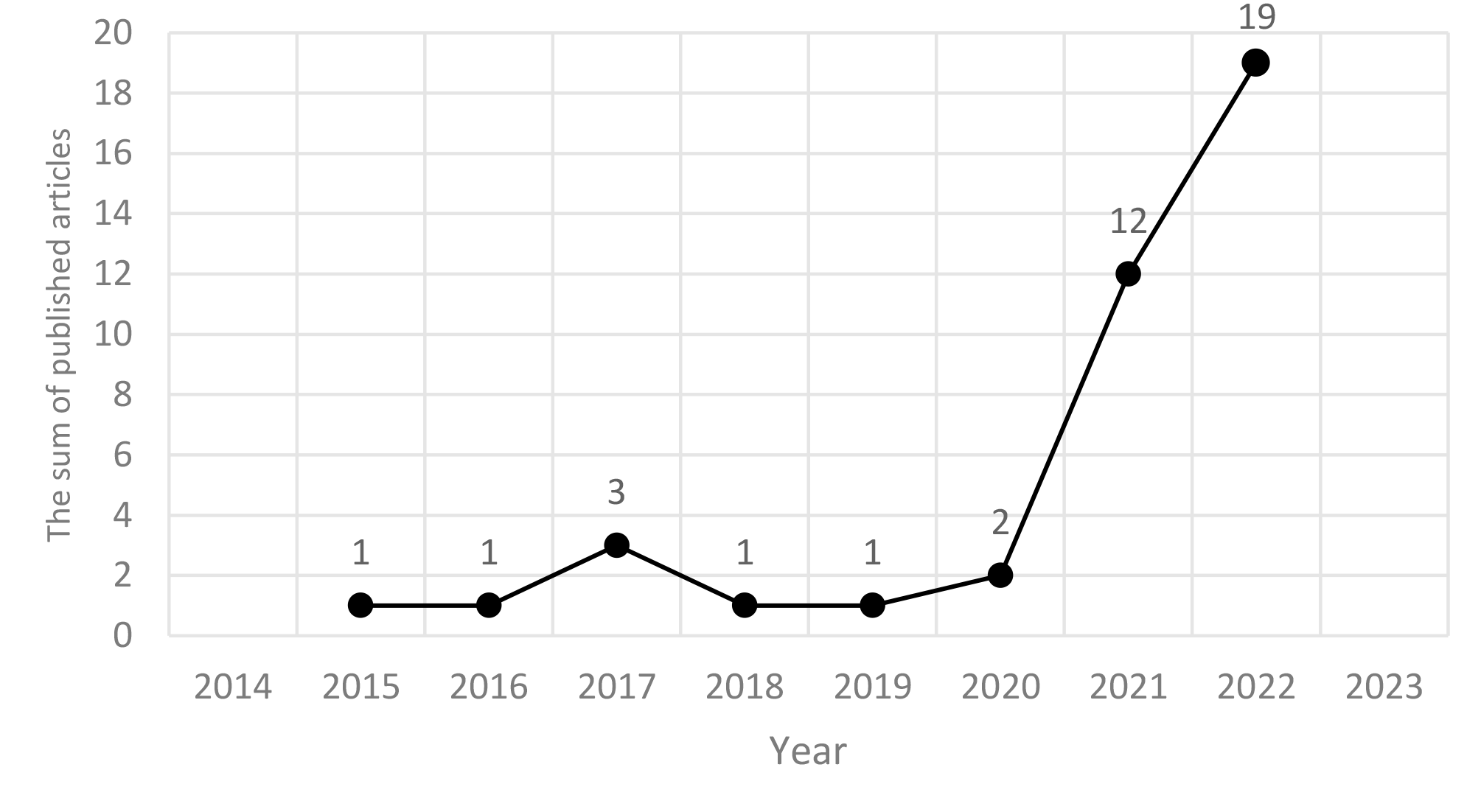


Figure S1: The trend of published articles (articles included)

Table S1: PRISMA 2020 checklists

| **Section and Topic** | **Item #** | **Checklist item** | **Location where item is reported** |
| --- | --- | --- | --- |
| **TITLE** | | |  |
| Title | 1 | Identify the report as a systematic review. | Page1 |
| **ABSTRACT** | | |  |
| Abstract | 2 | See the PRISMA 2020 for Abstracts checklist. | Page1 |
| **INTRODUCTION** | | |  |
| Rationale | 3 | Describe the rationale for the review in the context of existing knowledge. | Page2 |
| Objectives | 4 | Provide an explicit statement of the objective(s) or question(s) the review addresses. | Page2 |
| **METHODS** | | |  |
| Eligibility criteria | 5 | Specify the inclusion and exclusion criteria for the review and how studies were grouped for the syntheses. | Page2 |
| Information sources | 6 | Specify all databases, registers, websites, organisations, reference lists and other sources searched or consulted to identify studies. Specify the date when each source was last searched or consulted. | Page3 |
| Search strategy | 7 | Present the full search strategies for all databases, registers and websites, including any filters and limits used. | Page3 |
| Selection process | 8 | Specify the methods used to decide whether a study met the inclusion criteria of the review, including how many reviewers screened each record and each report retrieved, whether they worked independently, and if applicable, details of automation tools used in the process. | Page3 |
| Data collection process | 9 | Specify the methods used to collect data from reports, including how many reviewers collected data from each report, whether they worked independently, any processes for obtaining or confirming data from study investigators, and if applicable, details of automation tools used in the process. | Page3 |
| Data items | 10a | List and define all outcomes for which data were sought. Specify whether all results that were compatible with each outcome domain in each study were sought (e.g. for all measures, time points, analyses), and if not, the methods used to decide which results to collect. | Page3 |
|  | 10b | List and define all other variables for which data were sought (e.g. participant and intervention characteristics, funding sources). Describe any assumptions made about any missing or unclear information. | Page3-4 |
| Study risk of bias assessment | 11 | Specify the methods used to assess risk of bias in the included studies, including details of the tool(s) used, how many reviewers assessed each study and whether they worked independently, and if applicable, details of automation tools used in the process. | Page3-4 |
| Effect measures | 12 | Specify for each outcome the effect measure(s) (e.g. risk ratio, mean difference) used in the synthesis or presentation of results. | Page3-4 |
| Synthesis methods | 13a | Describe the processes used to decide which studies were eligible for each synthesis (e.g. tabulating the study intervention characteristics and comparing against the planned groups for each synthesis (item #5)). | Page3-4 |
|  | 13b | Describe any methods required to prepare the data for presentation or synthesis, such as handling of missing summary statistics, or data conversions. | Page3-4 |
|  | 13c | Describe any methods used to tabulate or visually display results of individual studies and syntheses. | Page3-4 |
|  | 13d | Describe any methods used to synthesize results and provide a rationale for the choice(s). If meta-analysis was performed, describe the model(s), method(s) to identify the presence and extent of statistical heterogeneity, and software package(s) used. | Page3-4 |
|  | 13e | Describe any methods used to explore possible causes of heterogeneity among study results (e.g. subgroup analysis, meta-regression). | Page3-4 |
|  | 13f | Describe any sensitivity analyses conducted to assess robustness of the synthesized results. | Page3-4 |
| Reporting bias assessment | 14 | Describe any methods used to assess risk of bias due to missing results in a synthesis (arising from reporting biases). | Page3-4 |
| Certainty assessment | 15 | Describe any methods used to assess certainty (or confidence) in the body of evidence for an outcome. | Page3-4 |
| **RESULTS** | | |  |
| Study selection | 16a | Describe the results of the search and selection process, from the number of records identified in the search to the number of studies included in the review, ideally using a flow diagram. | Page4 |
|  | 16b | Cite studies that might appear to meet the inclusion criteria, but which were excluded, and explain why they were excluded. | Page4 |
| Study characteristics | 17 | Cite each included study and present its characteristics. | Page4 |
| Risk of bias in studies | 18 | Present assessments of risk of bias for each included study. | Page4-8 |
| Results of individual studies | 19 | For all outcomes, present, for each study: (a) summary statistics for each group (where appropriate) and (b) an effect estimate and its precision (e.g. confidence/credible interval), ideally using structured tables or plots. | Page4-8 |
| Results of syntheses | 20a | For each synthesis, briefly summarise the characteristics and risk of bias among contributing studies. | Page4-8 |
|  | 20b | Present results of all statistical syntheses conducted. If meta-analysis was done, present for each the summary estimate and its precision (e.g. confidence/credible interval) and measures of statistical heterogeneity. If comparing groups, describe the direction of the effect. | Page4-8 |
|  | 20c | Present results of all investigations of possible causes of heterogeneity among study results. | Page4-8 |
|  | 20d | Present results of all sensitivity analyses conducted to assess the robustness of the synthesized results. | Page4-8 |
| Reporting biases | 21 | Present assessments of risk of bias due to missing results (arising from reporting biases) for each synthesis assessed. | Page4-8 |
| Certainty of evidence | 22 | Present assessments of certainty (or confidence) in the body of evidence for each outcome assessed. | Page4-8 |
| **DISCUSSION** | | |  |
| Discussion | 23a | Provide a general interpretation of the results in the context of other evidence. | Page8 |
|  | 23b | Discuss any limitations of the evidence included in the review. | Page8-9 |
|  | 23c | Discuss any limitations of the review processes used. | Page8-9 |
|  | 23d | Discuss implications of the results for practice, policy, and future research. | Page8-9 |
| **OTHER INFORMATION** | | |  |
| Registration and protocol | 24a | Provide registration information for the review, including register name and registration number, or state that the review was not registered. | Page2 |
|  | 24b | Indicate where the review protocol can be accessed, or state that a protocol was not prepared. | Page2 |
|  | 24c | Describe and explain any amendments to information provided at registration or in the protocol. | Page2 |
| Support | 25 | Describe sources of financial or non-financial support for the review, and the role of the funders or sponsors in the review. | Page10 |
| Competing interests | 26 | Declare any competing interests of review authors. | Page10 |

Table S2: Literature search strategy

**1.Pubmed**

| Search number | Query | Results |
| --- | --- | --- |
| #1 | "atrial fibrillation"[MeSH Terms] | 68457 |
| #2 | "atrial fibrillation"[Title/Abstract] OR "atrial fibrillations"[Title/Abstract] OR "atrium fibrillation"[Title/Abstract] OR "auricular fibrilation"[Title/Abstract] OR "auricular fibrillation"[Title/Abstract] OR "auricular fibrillations"[Title/Abstract] OR (("heart"[MeSH Terms] OR "heart"[All Fields] OR "hearts"[All Fields] OR "heart s"[All Fields]) AND "fibrillation atrium"[Title/Abstract]) | 89807 |
| #3 | "machine learning"[MeSH Terms] | 52598 |
| #4 | "machine learning"[Title/Abstract] OR "Adaboost"[Title/Abstract] OR "AlexNet"[Title/Abstract] OR "artificial intelligence"[Title/Abstract] OR "decision tree"[Title/Abstract] OR "deep learning"[Title/Abstract] OR "ensemble learning"[Title/Abstract] OR "GBM"[Title/Abstract] OR "gradient boosting"[Title/Abstract] OR "k nearest neighbors"[Title/Abstract] OR "naive bayes"[Title/Abstract] OR "neural network"[Title/Abstract] OR "Nomogram"[Title/Abstract] OR "prediction model"[Title/Abstract] OR "Radiomic"[Title/Abstract] OR "Radiomics"[Title/Abstract] OR "random forest"[Title/Abstract] OR "ResNet-50"[Title/Abstract] OR "ResNet101"[Title/Abstract] OR "ResNet18"[Title/Abstract] OR "ResNet-50"[Title/Abstract] OR "Risk-Prediction"[Title/Abstract] OR "Risk-Prediction"[Title/Abstract] OR "support vector machine"[Title/Abstract] OR "SVM"[Title/Abstract] OR "transfer learning"[Title/Abstract] OR "XGBoost"[Title/Abstract] | 256478 |
| #5 | "recurrence"[MeSH Terms] | 198821 |
| #6 | "Recurrence"[Title/Abstract] OR "Recrudescence"[Title/Abstract] OR "Recrudescences"[Title/Abstract] OR "Recurrences"[Title/Abstract] OR "Relapse"[Title/Abstract] OR "Relapses"[Title/Abstract] | 521379 |
| #7 | "atrial fibrillation"[MeSH Terms] OR ("atrial fibrillation"[Title/Abstract] OR "atrial fibrillations"[Title/Abstract] OR "atrium fibrillation"[Title/Abstract] OR "auricular fibrilation"[Title/Abstract] OR "auricular fibrillation"[Title/Abstract] OR "auricular fibrillations"[Title/Abstract] OR (("heart"[MeSH Terms] OR "heart"[All Fields] OR "hearts"[All Fields] OR "heart s"[All Fields]) AND "fibrillation atrium"[Title/Abstract])) | 101373 |
| #8 | "machine learning"[MeSH Terms] OR "machine learning"[Title/Abstract] OR "Adaboost"[Title/Abstract] OR "AlexNet"[Title/Abstract] OR "artificial intelligence"[Title/Abstract] OR "decision tree"[Title/Abstract] OR "deep learning"[Title/Abstract] OR "ensemble learning"[Title/Abstract] OR "GBM"[Title/Abstract] OR "gradient boosting"[Title/Abstract] OR "k nearest neighbors"[Title/Abstract] OR "naive bayes"[Title/Abstract] OR "neural network"[Title/Abstract] OR "Nomogram"[Title/Abstract] OR "prediction model"[Title/Abstract] OR "Radiomic"[Title/Abstract] OR "Radiomics"[Title/Abstract] OR "random forest"[Title/Abstract] OR "ResNet-50"[Title/Abstract] OR "ResNet101"[Title/Abstract] OR "ResNet18"[Title/Abstract] OR "ResNet-50"[Title/Abstract] OR "Risk-Prediction"[Title/Abstract] OR "Risk-Prediction"[Title/Abstract] OR "support vector machine"[Title/Abstract] OR "SVM"[Title/Abstract] OR "transfer learning"[Title/Abstract] OR "XGBoost"[Title/Abstract] | 261814 |
| #9 | "Recurrence"[MeSH Terms] OR "Recurrence"[Title/Abstract] OR "Recrudescence"[Title/Abstract] OR "Recrudescences"[Title/Abstract] OR "Recurrences"[Title/Abstract] OR "Relapse"[Title/Abstract] OR "Relapses"[Title/Abstract] | 640104 |
| #10 | ("atrial fibrillation"[MeSH Terms] OR ("atrial fibrillation"[Title/Abstract] OR "atrial fibrillations"[Title/Abstract] OR "atrium fibrillation"[Title/Abstract] OR "auricular fibrilation"[Title/Abstract] OR "auricular fibrillation"[Title/Abstract] OR "auricular fibrillations"[Title/Abstract] OR (("heart"[MeSH Terms] OR "heart"[All Fields] OR "hearts"[All Fields] OR "heart s"[All Fields]) AND "fibrillation atrium"[Title/Abstract]))) AND ("machine learning"[MeSH Terms] OR ("machine learning"[Title/Abstract] OR "Adaboost"[Title/Abstract] OR "AlexNet"[Title/Abstract] OR "artificial intelligence"[Title/Abstract] OR "decision tree"[Title/Abstract] OR "deep learning"[Title/Abstract] OR "ensemble learning"[Title/Abstract] OR "GBM"[Title/Abstract] OR "gradient boosting"[Title/Abstract] OR "k nearest neighbors"[Title/Abstract] OR "naive bayes"[Title/Abstract] OR "neural network"[Title/Abstract] OR "Nomogram"[Title/Abstract] OR "prediction model"[Title/Abstract] OR "Radiomic"[Title/Abstract] OR "Radiomics"[Title/Abstract] OR "random forest"[Title/Abstract] OR "ResNet-50"[Title/Abstract] OR "ResNet101"[Title/Abstract] OR "ResNet18"[Title/Abstract] OR "ResNet-50"[Title/Abstract] OR "Risk-Prediction"[Title/Abstract] OR "Risk-Prediction"[Title/Abstract] OR "support vector machine"[Title/Abstract] OR "SVM"[Title/Abstract] OR "transfer learning"[Title/Abstract] OR "XGBoost"[Title/Abstract])) AND ("Recurrence"[MeSH Terms] OR ("Recurrence"[Title/Abstract] OR "Recrudescence"[Title/Abstract] OR "Recrudescences"[Title/Abstract] OR "Recurrences"[Title/Abstract] OR "Relapse"[Title/Abstract] OR "Relapses"[Title/Abstract])) | 115 |

**2.Cochrane**

| Search number | Query | Results |
| --- | --- | --- |
| #1 | MeSH descriptor: [Atrial Fibrillation] explode all trees | 5292 |
| #2 | (Atrial Fibrillation):ti,ab,kw OR (Atrial Fibrillations):ti,ab,kw OR (atrium fibrillation):ti,ab,kw OR (auricular fibrilation):ti,ab,kw OR (Auricular Fibrillation):ti,ab,kw | 15198 |
| #3 | (Auricular Fibrillations):ti,ab,kw OR (heart fibrillation atrium):ti,ab,kw | 2520 |
| #4 | #1 or #2 or #3 | 15198 |
| #5 | MeSH descriptor: [Machine Learning] explode all trees | 295 |
| #6 | (machine learning):ti,ab,kw OR (Adaboost):ti,ab,kw OR (AlexNet):ti,ab,kw OR (artificial intelligence):ti,ab,kw OR (Decision tree):ti,ab,kw | 4089 |
| #7 | (Deep Learning):ti,ab,kw OR (ensemble learning):ti,ab,kw OR (GBM):ti,ab,kw OR (Gradient Boosting):ti,ab,kw OR (K-Nearest Neighbors):ti,ab,kw | 2692 |
| #8 | (Naive Bayes):ti,ab,kw OR (neural network):ti,ab,kw OR (Nomogram):ti,ab,kw OR (prediction model):ti,ab,kw OR (Radiomic):ti,ab,kw | 8452 |
| #9 | (Radiomics):ti,ab,kw OR (random forest):ti,ab,kw OR (ResNet 50):ti,ab,kw OR (ResNet101):ti,ab,kw OR (ResNet18):ti,ab,kw | 1146 |
| #10 | (ResNet-50):ti,ab,kw OR (Risk Prediction):ti,ab,kw OR (Risk-Prediction):ti,ab,kw OR (Support Vector Machine):ti,ab,kw OR (SVM):ti,ab,kw | 6300 |
| #11 | (Transfer Learning):ti,ab,kw OR (XGBoost):ti,ab,kw | 1522 |
| #12 | #5 or #6 or #7 or #8 or #9 or #10 or #11 | 18084 |
| #13 | MeSH descriptor: [Recurrence] explode all trees | 12920 |
| #14 | (Recurrence):ti,ab,kw OR (Recrudescence):ti,ab,kw OR (Recrudescences):ti,ab,kw OR (Recurrences):ti,ab,kw OR (Relapse):ti,ab,kw | 79773 |
| #15 | (Relapses):ti,ab,kw | 5750 |
| #16 | #13 or #14 or #15 | 81355 |
| #17 | #4 and #12 and #16 | 35 |

**3.Embase**

| Search number | Query | Results |
| --- | --- | --- |
| #1 | 'atrial fibrillation'/exp | 201068 |
| #2 | 'atrial fibrillation':ab,ti OR 'atrial fibrillations':ab,ti OR 'atrium fibrillation':ab,ti OR 'auricular fibrilation':ab,ti OR 'auricular fibrillation':ab,ti OR 'auricular fibrillations':ab,ti OR 'heart fibrillation atrium':ab,ti | 154771 |
| #3 | #1 OR #2 | 213799 |
| #4 | 'machine learning'/exp | 356450 |
| #5 | 'machine learning':ab,ti OR adaboost:ab,ti OR alexnet:ab,ti OR 'artificial intelligence':ab,ti OR 'decision tree':ab,ti OR 'deep learning':ab,ti OR 'ensemble learning':ab,ti OR gbm:ab,ti OR 'gradient boosting':ab,ti OR 'k-nearest neighbors':ab,ti OR 'naive bayes':ab,ti OR 'neural network':ab,ti OR nomogram:ab,ti OR 'prediction model':ab,ti OR radiomic:ab,ti OR radiomics:ab,ti OR 'random forest':ab,ti OR resnet101:ab,ti OR resnet18:ab,ti OR 'resnet 50':ab,ti OR 'risk prediction':ab,ti OR 'support vector machine':ab,ti OR svm:ab,ti OR 'transfer learning':ab,ti OR xgboost:ab,ti | 310932 |
| #6 | #4 OR #5 | 510264 |
| #7 | recurrence:ab,ti OR recrudescence:ab,ti OR recrudescences:ab,ti OR recurrences:ab,ti OR relapse:ab,ti OR relapses:ab,ti | 812956 |
| #8 | #3 AND #6 AND #7 | 211 |

**4.Web of science**

| Search number | Query | Results |
| --- | --- | --- |
| #1 | Atrial Fibrillation (Topic) OR Atrial Fibrillations (Topic) OR atrium fibrillation (Topic) OR auricular fibrilation (Topic) OR Auricular Fibrillation (Topic) OR Auricular Fibrillations (Topic) OR heart fibrillation atrium (Topic) | 121494 |
| #2 | machine learning (Topic) OR Adaboost (Topic) OR AlexNet (Topic) OR artificial intelligence (Topic) OR Decision tree (Topic) OR Deep Learning (Topic) OR ensemble learning (Topic) OR GBM (Topic) OR Gradient Boosting (Topic) OR K-Nearest Neighbors (Topic) OR Naive Bayes (Topic) OR neural network (Topic) OR Nomogram (Topic) OR prediction model (Topic) OR Radiomic (Topic) OR Radiomics (Topic) OR random forest (Topic) OR ResNet 50 (Topic) OR ResNet101 (Topic) OR ResNet18 (Topic) OR ResNet-50 (Topic) OR Risk Prediction (Topic) OR Risk-Prediction (Topic) OR Support Vector Machine (Topic) OR SVM (Topic) OR Transfer Learning (Topic) OR XGBoost (Topic | 2018214 |
| #3 | Recurrence (Topic) OR Recrudescence (Topic) OR Recrudescences (Topic) OR Recurrences (Topic) OR Relapse (Topic) OR Relapses (Topic) | 599753 |
| #4 | #1 AND #2 AND #3 | 409 |

Table S3: Baseline characteristics of the 40 studies selected for the meta-analysis

| No. | First author | Year of publication | Author country | Study type | Patient source | Atrial Fibrillation Recurrence Monitoring Device | Modeling variables | Definition of AF Recurrence | Ablation type | Blanking period | Median length of follow-up | Maximum follow-up |
| --- | --- | --- | --- | --- | --- | --- | --- | --- | --- | --- | --- | --- |
| 1 | Xue Zhou | 2022 | Japan | Retrospective cohort study | Single center | 12-lead ECG or Holter or Implantable loop recorder | NT-proBNP, left atrial volume , left atrial appendage volume， type of AF,AF duration，Right atrial volume，Left Atrial Diameter | Any atrial arrhythmia (e.g., AF, atrial flutter, or atrial tachycardia) lasting >30 seconds is recorded 3 months after ablation. | RFCA | 3m | 13.5m | 24m |
| 2 | Zhihao Zhao | 2022 | China | Retrospective cohort study | Single center | 12-lead ECG or Holter. | left atrial diameter , left ventricular ejection fraction , type of AF, systemic inflammation score | Any atrial arrhythmia (e.g., AF or atrial flutter or atrial tachycardia) lasting >30 seconds is recorded 3 months after ablation. | RFCA | 3m | 25m | N/A |
| 3 | Min Yang | 2022 | China | Retrospective cohort study | Single center | 12-lead ECG or Holter. | Radiomics features | Any atrial arrhythmia (e.g., AF, atrial flutter, or atrial tachycardia) lasting >30 seconds is recorded within 3 months to 1 year after ablation. | CBCA | 3m | N/A | 12m |
| 4 | Siyi Tang | 2022 | America | Retrospective cohort study | Multicenter | Implantable loop recorders or 12-lead ECG | left ventricular ejection fraction, height, BMI, weight, left atria volume , left atria surface area，Radiomics features | AF recurrence was defined as >30 second duration episodes on ECG  monitoring, or >1% AF burden on device interrogation for the  patients with implantable monitor | CACA+RFCA | N/A | N/A | 12m |
| 5 | Weiping Sun | 2022 | china | Prospective cohort study | Single center | 12-lead ECG or Holter. | tissue inhibitor of metalloproteinase-1 ，left atrium diameter | AF is recorded 3 months after ablation. | RFCA | 3m | N/A | 12m |
| 6 | Jingyu Sheng MD | 2022 | china | Retrospective cohort study | Multicenter | 12-lead ECG or Holter. | age,  degrees of mitral regurgitation, left atrial appendage  emptying velocity | Any atrial arrhythmia (e.g., AF, atrial flutter, or atrial tachycardia) lasting ≥30 s is recorded 3 months after ablation. | RFCA | 3m | N/A | 6m |
| 7 | Andrea Saglietto | 2022 | Italy | Prospective cohort study | Multicenter | 12-lead ECG or Holter or Implantable loop recorders | left ventricular end-diastolic volume，Estimated glomerular filtration rate，BMI， Age，left  atrium anteroposterior diameter，left ventricular  ejection fraction，CHAD2DS2-VASc score，Dyslipidaemia， Atrial fibrillation of type，Structural heart disease，Hypertension，Gender， Heart failure，coronary artery disease，Smoker | Any atrial arrhythmia (e.g., atrial fibrillation, atrial flutter, or atrial tachycardia) lasting ≥30 s is recorded within 3 months to 1 year after ablation. | CA | 3m | 12.4m | N/A |
| 8 | Zhong-bao Ruan | 2022 | china | Retrospective cohort study | Single center | 12-lead ECG or Holter | Monocyte count/high-density lipoprotein  cholesterol， AF course， coronary heart disease ，Type of atrial fibrillation，Fatty Acid-Binding Protein 4 Type of af | Any atrial arrhythmia (e.g., AF or atrial flutter or atrial tachycardia) lasting ≥30 s is recorded 3 months after ablation. | RFCA | 3m | 12.02 m | N/A |
| 9 | Caroline H. Roney | 2022 | UK | Retrospective cohort study | Single center | Holter | left ventricular ejection fraction, body mass  index, age, female sex, congestive heart failure, history of hypertension, diabetes, history of  stroke, coronary disease, type of AF，Radiomics features | N/A | RFCA | 3m | N/A | 12m |
| 10 | José Nicolás López-Canoa | 2022 | Spain | Retrospective cohort study | Single center | 12-lead ECG or Holter | albumin‐to‐globulin ratio，red blood cell distribution width，CHADS2 | Any atrial arrhythmia (e.g., AF, atrial flutter, or atrial tachycardia) lasting >30 seconds is recorded 3 months after ablation | CA | 3m | 32.4m | N/A |
| 11 | Yu Liu | 2022 | china | Retrospective cohort study | Single center | 12‐lead ECG or Holter | "left ventricular ejection fraction, body mass  index, age, female sex, congestive heart failure, history of hypertension, diabetes, history of  stroke, coronary disease, and atrial fibrillation type，Radiomics features" | Any atrial fibrillation lasting ≥30 seconds is recorded within 3 months to 1 year after ablation. | CBCA | 3m | 19m | N/A |
| 12 | Zhitong Li | 2022 | china | Retrospective cohort study | Single center | ECG or Holter | Type of atrial fibrillation，Fatty Acid-Binding Protein 4 | Any AF or atrial tachycardia lasting ≥30 seconds is recorded 3 months after ablation. | RFCA | 3m | 35m | N/A |
| 13 | Guangling Li | 2022 | china | Retrospective cohort study | Single center | 12-lead ECG  or Holter | albumin‐to‐globulin ratio，red blood cell distribution width，CHADS2 | 1. Any atrial arrhythmia (e.g., AF, atrial flutter, or atrial tachycardia) lasting >30 seconds is recorded, within 3 months to 1 year after ablation. 2.patients who still could not stop AADs after  blank period are considered to have a AF recurrence. | RFCA | N/A | N/A | 12m |
| 14 | Dae-In Lee | 2022 | Korea | Retrospective cohort study | Single center | ECG or Holter | Coronary heart disease; left atrial diameter; Age; Types of antiarrhythmic drugs that fail treatment; Female; AF duration, type of atrial fibrillation | Within 1 year after ablation, atrial flutter,  atrial tachycardia or AF lasting more than 30 seconds is recorded on Holter ECG  or more than 10 seconds on a 12-lead ECG. | RFCA | 3m | N/A | 12m |
| 15 | Daniel J. Lauritzen | 2022 | Denmark | Retrospective cohort study | Single center | 12-lead ECG  or Holter | age, coronary  artery disease , heart failure , hypertension, transient ischemic attack  or cerebrovascular accident 、 left atrial diameter" | Between 3 months and 1 year after ablation, AF is recorded for at least 30 seconds according to  consistent statements defined by current. | RFCA | 3m | N/A | 12m |
| 16 | Michael A LaBarbera | 2022 | America | Retrospective cohort study | Single center | NA | age,  sex, height, weight, hypertension, AF of type, AF duration, Left Atrial Diameter , left ventricular mass index, eGFR" | Any atrial arrhythmia (e.g., AF, atrial flutter, or atrial tachycardia) lasting >30 seconds is recorded 3 months after ablation. | CBCA+RFCA | 3m | N/A | 14m |
| 17 | Sixiang Jia | 2022 | china | Retrospective cohort study | Single center | 12-lead ECG or Holter | gender  Left Ventricular Ejection Fractions,  hypertension ， age ，  diabetes ， previous stroke/transient ischemic attack" | AF recurrence is defined as an AF episode. | RFCA | N/A | N/A | 12m |
| 18 | Wenqiang Han | 2022 | china | Prospective cohort study | Single center | Holter or ECG | age, BMI, his-  tory of Apixaban use  ，Radiomics features" | Any AF, atrial flutter, or atrial tachycardia lasting >30 seconds is recorded 3 months after ablation. | CA | 3m | N/A | 24m |
| 19 | Youzheng Dong | 2022 | china | Prospective cohort study | Single center | Holter or ECG | age ， snoring ，BMI ，AF history ，hypertension ，coronary heart disease ，diabetes ，heart failure ，valve diseases ， cardiomyopathy ， Left Atrial Diameter ， strok，gender | Any AF, atrial flutter, or atrial tachycardia lasting >30 seconds is recorded 3 months after ablation. | RFCA | 3m | 17.2m | 43m |
| 20 | Xiyu Zhu | 2021 | china | Retrospective cohort study | Single center | Holter | MI, duration of AF history, gender, responses after using  nifekalant, left atrial diameter" | NA | RFCA | N/A | 19.8m | 12m |
| 21 | Xiao-Juan Zhou | 2021 | china | Prospective cohort study | Single center | ECG or Holter | age , AF duration,  circ 81906-RYR2 | Any atrial arrhythmia (e.g., AF, atrial flutter, or atrial tachycardia) lasting >30 seconds is recorded 3 months after ablation. | RFCA | 3m | N/A | 12m |
| 22 | Zhenni Yang | 2021 | china | Retrospective cohort study（） | Single center | ECG or Holter | brain natriuretic peptid; Diastolic emptying index; left atrial appendage peak emptying velocity | Any atrial arrhythmia (e.g., AF, atrial flutter, or atrial tachycardia) lasting >30 seconds is recorded 3 months after ablation. | RFCA | 3m | 4m | 6m |
| 23 | Aditi S. Vaishnav | 2021 | America | Retrospective cohort study | Database | Implantable loop recorders or ECG | Radiomics features | Any AF or atrial tachycardia for greater than or equal to 30s is recorded. | CBCA | 3m | N/A | 12m |
| 24 | Yuxia Miao MM | 2021 | china | Retrospective cohort study | Single center | Implantable loop recorders | age, type of AF ， hypertension ， ablation type，Radiomics features | Any AF for ≥ 2 minutes is recorded 3 months after ablation. | RFCA | N/A | N/A | 6m |
| 25 | Xin- Xin Ma | 2021 | China | Prospective cohort study | Single center | 12-lead ECG or Holter | Radiomics features，AF duration. | Any atrial arrhythmia (e.g., AF, atrial flutter, or premature atrial contraction) lasting >30 s is recorded 3 months after ablation. | RFCA | 3m | 12m | N/A |
| 26 | Takashi Kakuta | 2021 | Japan | Retrospective cohort study | Single center | 12- lead ECG or Holter | Type of AF, left atrial appendage emptying flow velocity, left Atrial maximal volumes index ， global longitudinal strain | AF or any other atrial tachyarrhythmia lasting at least 30 seconds | CBCA | N/A | 62m | N/A |
| 27 | Marek Jastrzębski | 2021 | Poland | Prospective cohort study | Single center | 12- lead ECG or Holter | F-wave voltage, AF duration, Preoperative Left atrial volume indexi, age | NA | CBCA | 3m | 28.6m | 120 |
| 28 | Yi-Ting Hwang | 2021 | china（Taiwan） | Retrospective cohort study | Single center | ECG or Holter or intracardiac electrogram | left atrial dimension，Type of atrial fibrillation | AF lasting 30 s is recorded 3 months after ablation. | RFCA | N/A | 6m | N/A |
| 29 | Fuqian Guo | 2021 | china | Retrospective cohort study | Single center | 12-lead ECG or Holter or Implantable loop recordersor | Radiomics features | Palpitation episode or atrial tachyarrhythmia >30 seconds | CA | 3m | N/A | N/A |
| 30 | Marjan Firouznia | 2021 | America | Retrospective cohort study | Single center | 12- lead ECG or Holter | left atrial minimum volume index， LA sphericity，CHA2DS2-VASc score | AF lasting 30 s is recorded 3 months after ablation | CA | N/A | N/A | N/A |
| 31 | Thomas Atta‑Fosu | 2021 | America | case-control study | Single center | NA | Radiomics features，Age，Sex ，Height ，Weight ，Hypertension，Diabetes ，Left Ventricular Ejection Fraction， Type of  AF ，Ablation Energy Type ，coronary artery disease | NA | - CBCA+RFCA | 3m | N/A | N/A |
| 32 | Na Yang | 2020 | china | Retrospective cohort study | Single center | ECG or Holter | age, sex, left atrial volume, left ventricular ejection fraction, BMI ，sinus rhythm, Type of  AF ，ablation type，Radiomics features | NA | RFCA | 3m | 12m | N/A |
| 33 | Graham Peigh | 2020 | America | Retrospective cohort study | Single center | 12-lead  ECG or Holter | Coronary artery disease，Left atrial diameter， Age， Type of  AF，Antiarrhythmics failed， gender，P-wave duration，Voltage in lead I， | Atrial arrhythmia for more than 30 seconds is recorded between 3 months and 12 months after ablation. | CBCA | 3m | 33m | N/A |
| 34 | Tatjana S Potpara | 2019 | serbia | Retrospective cohort study | Single center | ECG or  Implantable loop recorders or Kardia smartphone monitors | Structural heart disease ，Coronary artery disease ，left Atrial diameter， Left bundle branch block，Early return of AF， Type of AF | Atrial arrhythmia for more than 30 seconds is recorded within 3 months to 12 months after AF ablation. | CA | 3m | 24m | N/A |
| 35 | Yemei He | 2018 | china | Retrospective cohort study | Single center | 12-lead  ECG or Holter | gender ，bundle branch block; early  recurrence of AF，  coronary artery disease, left atrial diameter, age, Type of  AF, antiarrhythmic drugs failed,  hypertension, diabetes mellitus, prior stroke，congestive heart failure or left ventricular systolic dysfunction, | Atrial arrhythmia lasting more than 30 seconds is recorded within 3 months to 12 months after AF ablation. | RFCA | 3m | N/A | 12m |
| 36 | Mohamed Sanhoury | 2017 | Italy | Retrospective cohort study | Single center | 12-lead  ECG or Holter | left atrial volume index ， left atrial appendage ejection fraction， left atrial  appendage peak emptying flow velocity" | Atrial tachyarrhythmias (AF, atrial tachycardia, atrial flutter) lasting > 30  seconds are recorded 3 months after AF ablation. | CBCA | 3m | 18m | N/A |
| 37 | Nebojša Mujović | 2017 | Serbia | Retrospective cohort study | Single center | Implantable loop recordersor 12-lead ECG or Holter | Coronary artery disease ,Left atrial diameter ,Age, Type of  AF,Number of previously failed AAD, gender | Atrium  tachyarrhythmia (AF, AT, and/or AFL) lasting >30s is recorded 3 months after AF ablation. | RFCA | 3m | 29.1m | 36m |
| 38 | Shuman Jia | 2017 | France | case-control study | Single center | 12-lead ECG or Holter | gender, Bundle brunch block, Left atrium Type of  AF，early recurrent AF | Atrial tachyarrhythmia (AF, atrial tachycardia, atrial flutter) lasting > 30 seconds after ablation | CA | N/A | N/A | N/A |
| 39 | Vicente Zarzoso | 2016 | France | Retrospective cohort study | Single center | 12-lead ECG or Holter | I,V1,V2,V5 leads f wave amplitude | AF or atrial flutter flutter > 30 seconds is recorded. | RFCA | 3m | 13.9m | N/A |
| 40 | Jelena Kornej | 2015 | Germany | Retrospective cohort study | Multicenter | Holter | age， Type of AF，eGFR ，LA diameter，EF | 1. Any atrial arrhythmia lasting ≥ 30s occurs within 3 months to 1 year after ablation, or antiarrhythmic drugs need to be taken to maintain sinus rhythm 3 months after ablation. | CA | 3m | N/A | N/A |

**Table S3: Baseline characteristics of the 40 studies selected for the meta-analysis (continue)**

| No. | Number of recurrent cases | Total number of cases | Number of recurrent cases in the training set | Total number of cases in the training set | Generation of the validation set | Overfitting method | Number of recurrent cases in the validation set | Total number of cases in the validation set | Missing value processing method | Variable screening/feature selection method | Model type |
| --- | --- | --- | --- | --- | --- | --- | --- | --- | --- | --- | --- |
| 1 | 94 | 310 | 94 | 310 | 10-foldcross validation | 10-fold crossvalidation | N/A | N/A | Mean imputation | Univariate | CNNSurv/ DeepSurv/ Cox |
| 2 | 207 | 485 | 149 | 340 | - Random sampling | N/A | 58 | 145 | N/A | Univariate + multivariate Logistic | LR |
| 3 | 79 | 314 | 68 | 251 | Random sampling +10- fold cross validation | 10- fold cross validation | 11 | 63 | No missing value | Univariate + multivariate Logistic | LR |
| 4 | 44 | 156 | 44 | 156 | 10- fold cross validation | 10- fold cross validation | N/A | N/A | Mean imputation and median imputation | Variable importance | XGBoost/CNN |
| 5 | 72 | 230 | 72 | 230 | 10- fold cross validation | 10- fold cross validation +bootstrap | N/A | N/A | N/A | Univariate + multivariate Logistic | LR |
| 6 | 24 | 276 | 18 | 207 | Random sampling | N/A | 6 | 69 | N/A | Univariate + multivariate Logistic | LR |
| 7 | 797 | 3128 | 638 | 2502 | Random sampling +10-fold cross validation | 10-fold cross validation | 159 | 626 | N/A | Variable importance | DT/ RF/ AdaBoost /kNN |
| 8 | 59 | 221 | 59 | 221 | N/A | N/A | N/A | N/A | N/A | Univariate + multivariate  COX | COX |
| 9 | 34 | 99 | 34 | 99 | 10-fold cross validation | 10-fold cross validation | N/A | N/A | No missing value | N/A | SVM/KNN/ RF/LR |
| 10 | 188 | 299 | 188 | 299 | N/A | N/A | N/A | N/A | N/A | N/A | COX |
| 11 | 35 | 84 | 35 | 84 | N/A | N/A | N/A | N/A | N/A | Univariate + multivariate | COX |
| 12 | 643 | 1763 | 643 | 1763 | N/A | Bootstrap | N/A | N/A | N/A | No variable screening | COX |
| 13 | 134 | 433 | 96 | 305 | Random sampling +10- fold cross validation | 10- fold cross validation | 38 | 128 | Mean imputation | Univariate + multivariate | COX |
| 14 | 47 | 177 | 35 | 133 | 4-fold cross validation + random sampling | N/A | 12 | 44 | N/A | Variable importance | ANN/LR/XGBoost/SVM/MLP |
| 15 | 104 | 337 | 104 | 337 | N/A | N/A | N/A | N/A | N/A | Non variable screening | LR |
| 16 | 97 | 216 | 75 | 150 | External validation + 5-fold cross validation | 5-fold cross validation | 22 | 66 | No missing value | Variable importance | LDA |
| 17 | 39 | 200 | 27 | 140 | 10-fold cross validation + random sampling | 10-fold cross validation | 12 | 60 | N/A | LASSO | LR |
| 18 | 316 | 1065 | 220 | 710 | Random sampling | N/A | 96 | 355 | N/A | Univariate + multivariate | LR |
| 19 | 107 | 449 | 64 | 244 | External validation +10-fold cross validation | Bootstrap | 43 | 205 | flux-linkage equations, multiple imputation | Lasso | COX |
| 20 | 64 | 136 | 64 | 136 | N/A | N/A | N/A | N/A | N/A | Univariate + multivariate | LR |
| 21 | 79 | 312 | 79 | 312 | N/A | Bootstrap | N/A | N/A | N/A | Univariate + multivariate | LR |
| 22 | 55 | 215 | 55 | 215 | N/A | Bootstrap | N/A | N/A | N/A | Univariate + multivariate | LR |
| 23 | 11 | 58 | 11 | 58 | N/A | N/A | N/A | N/A | N/A | Univariate + multivariate | LR |
| 24 | N/A | 403 | 39 | 206 | Random sampling +10-fold cross validation | 10-fold cross validation | N/A | 197 | No missing value | Lasso | LR |
| 25 | 41 | 128 | 41 | 128 | N/A | bootstrap | N/A | N/A | N/A | Univariate + multivariate | LR |
| 26 | 257 | 450 | 154 | 270 | Random sampling | N/A | 103 | 180 | Multiple imputation | multivariate | LR |
| 27 | 191 | 588 | 191 | 588 | N/A | N/A | N/A | N/A | N/A | Univariate + multivariate | COX |
| 28 | 163 | 606 | 70 | 260 | Random sampling | N/A | 18 | 66 | No missing value | N/A | CNN/LR |
| 29 | 30 | 105 | 30 | 105 | N/A | N/A | N/A | N/A | No missing value | Univariate + multivariate | LR |
| 30 | 88 | 203 | 68 | 137 | External validation | N/A | 20 | 66 | No missing value | Variable ordering | RF |
| 31 | 37 | 68 | 37 | 68 | 5- fold cross validation | 5- fold cross validation | N/A | N/A | No missing value | N/A | XGBoostt |
| 32 | 67 | 207 | 67 | 207 | N/A | N/A | N/A | N/A | N/A | multivariate | LR |
| 33 | 77 | 300 | 77 | 300 | N/A | N/A | N/A | N/A | N/A | Univariate + multivariate | LR |
| 34 | 133 | 226 | 133 | 226 | N/A | N/A | N/A | N/A | N/A | Univariate | COX |
| 35 | 24 | 80 | 24 | 80 | N/A | N/A | N/A | N/A | No missing value | Univariate + multivariate | LR |
| 36 | 25 | 283 | 25 | 283 | N/A | N/A | N/A | N/A | N/A | No variable screening | LR |
| 37 | 20 | 133 | 20 | 133 | N/A | N/A | 3 | 39 | N/A | Univariate | COX |
| 38 | 13 | 40 | 13 | 40 | N/A | N/A | N/A | N/A | No missing value | Partial least squares regression | COX |
| 39 | 15 | 62 | 15 | 62 | N/A | N/A | N/A | N/A | N/A | Univariate + multivariate | LR |
| 40 | 538 | 1406 | 377 | 1145 | N/A | N/A | 185 | 261 | N/A | N/A | LR |

Table S4: Risk of bias and applicability assessment by PROBAST criteria

| **No** | **Study** | **Model** | **RoB Participants** | **RoB Predictors** | **RoB Outcome** | **RoB Analysis** | **Overall RoB** |
| --- | --- | --- | --- | --- | --- | --- | --- |
| 1 | Zhou 2022 | Cox | L | L | U | H | H |
|  |  | DeepSurv | L | L | U | H | H |
|  |  | CNNSurv | L | L | U | H | H |
| 2 | Zhao 2022 | LR | L | L | U | U | U |
| 3 | Yang 2022 | LR | L | L | U | H | H |
| 4 | Tang 2022 | XGBoost | L | L | U | H | H |
|  |  | CNN | L | L | U | H | H |
| 5 | Sun 2022 | LR | L | L | L | H | H |
| 6 | Sheng 2022 | LR | L | L | U | H | H |
| 7 | Saglietto 2022 | DT | L | L | L | U | U |
|  |  | KNN | L | L | L | U | U |
|  |  | AdaBoost | L | L | L | U | U |
|  |  | RF | L | L | L | U | U |
| 8 | Ruan 2022 | Cox | L | L | U | H | H |
| 9 | Roney 2022 | RF | L | L | U | H | H |
|  |  | KNN | L | L | U | H | H |
|  |  | SVM | L | L | U | H | H |
|  |  | LR | L | L | U | H | H |
| 10 | Canoa 2022 | COX | L | L | U | H | H |
| 11 | Liu 2022 | COX | L | L | U | H | H |
| 12 | Li 2022 | COX | L | L | U | U | U |
| 13 | Li 2022 | COX | L | L | U | H | H |
| 14 | Lee 2022 | ANN | L | L | U | H | H |
|  |  | LR | L | L | U | H | H |
|  |  | XGBoost | L | L | U | H | H |
|  |  | SVM | L | L | U | H | H |
|  |  | MLP | L | L | U | H | H |
| 15 | Lauritzen 2022 | LR | L | L | U | H | H |
| 16 | LaBarbera 2022 | LDA | L | L | U | H | H |
| 17 | Jia 2022 | LR | L | L | U | H | H |
| 18 | Han 2022 | LR | L | L | L | H | H |
| 19 | Dong 2022 | COX | L | L | L | U | U |
| 20 | Zhu 2021 | LR | L | L | U | H | H |
| 21 | Zhou 2021 | LR | L | L | L | U | U |
| 22 | Yang 2021 | LR | L | L | U | U | U |
| 23 | Vaishnav 2021 | LR | L | L | U | H | H |
| 24 | MM 2021 | LR | L | L | U | U | U |
| 25 | Ma 2021 | LR | L | L | L | U | U |
| 26 | Kakuta 2021 | LR | L | L | U | H | H |
| 27 | Jastrzębski 2021 | COX | L | L | L | H | H |
| 28 | Hwang 2021 | CNN | L | L | U | H | H |
|  |  | LR | L | L | U | H | H |
| 29 | Guo 2021 | LR | L | L | U | H | H |
| 30 | Firouznia 2021 | RF | L | L | U | H | H |
| 31 | Atta‑Fosu 2021 | XGBoost | H | U | H | H | H |
| 32 | Yang 2020 | LR | L | L | U | H | H |
| 33 | Peigh 2020 | LR | L | L | U | H | H |
| 34 | Potpara 2019 | COX | L | L | U | H | H |
| 35 | He 2018 | LR | L | L | U | H | H |
| 36 | Sanhoury 2017 | LR | L | L | U | H | H |
| 37 | Mujović 2017 | COX | L | L | U | H | H |
| 38 | Jia 2017 | COX | H | U | H | H | H |
| 39 | Zarzoso 2016 | LR | L | L | U | H | H |
| 40 | Kornej 2015 | LR | L | L | U | H | H |
